# Cognitive Problem Solving Patterns of Medical Students Correlate with Success in Diagnostic Case Solutions

**DOI:** 10.1371/journal.pone.0071486

**Published:** 2013-08-12

**Authors:** Jan Kiesewetter, René Ebersbach, Anja Görlitz, Matthias Holzer, Martin R. Fischer, Ralf Schmidmaier

**Affiliations:** 1 Lehrstuhl für Didaktik und Ausbildungsforschung in der Medizin am Klinikum der Universität München, Ludwig-Maximilians-University, Munich, Germany; 2 Medizinische Klinik und Poliklinik IV, Klinikum der Universität München, Ludwig-Maximilians-University, Munich, Germany; Catholic University of Sacro Cuore, Italy

## Abstract

**Context:**

Problem-solving in terms of clinical reasoning is regarded as a key competence of medical doctors. Little is known about the general cognitive actions underlying the strategies of problem-solving among medical students. In this study, a theory-based model was used and adapted in order to investigate the cognitive actions in which medical students are engaged when dealing with a case and how patterns of these actions are related to the correct solution.

**Methods:**

Twenty-three medical students worked on three cases on clinical nephrology using the think-aloud method. The transcribed recordings were coded using a theory-based model consisting of eight different cognitive actions. The coded data was analysed using time sequences in a graphical representation software. Furthermore the relationship between the coded data and accuracy of diagnosis was investigated with inferential statistical methods.

**Results:**

The observation of all main actions in a case elaboration, including evaluation, representation and integration, was considered a *complete model* and was found in the majority of cases (56%). This pattern significantly related to the accuracy of the case solution (φ = 0.55; p<.001). Extent of prior knowledge was neither related to the *complete model* nor to the correct solution.

**Conclusions:**

The proposed model is suitable to empirically verify the cognitive actions of problem-solving of medical students. The cognitive actions *evaluation, representation* and *integration* are crucial for the *complete model* and therefore for the accuracy of the solution. The educational implication which may be drawn from this study is to foster students reasoning by focusing on higher level reasoning.

## Introduction

The physician's profession demands a number of competencies. One of these is the ability to reason clinically. Clinical reasoning focuses on the signs and symptoms of a patient and the subsequent identification of relevant questions on the patient´s history, further the physical examination, the correct interpretation of those results and information, as well as procedures required to reach the correct diagnosis in an efficient manner [1]. The actual reasoning process involves medical decision-making on the one hand and problem-solving on the other hand [2]. This study focuses on medical problem-solving. There is a broad base of knowledge on expertise of physicians and their decision-making (cf. [3]), but only little is known about cognitive actions of medical students. This lack of knowledge exacerbates attempts of medical educators to foster problem-solving adapted to their students’ needs. This study focuses therefore only on medical students. Prior knowledge is essential for successful problem-solving as shown by various studies regarding “content specificity” [4], [5]. Previous research has identified a spectrum of four consecutive strategies for problem-solving in medicine: guessing, hypothetical-deductive reasoning, scheme induction and pattern recognition [6]. With increasing knowledge and experience, medical students derive hypotheses from the patient’s information and try to verify them purposefully. These strategies of generating and testing of hypotheses have successfully been observed empirically[7]–[9] and described in detail[6], [10]–[12]. In the last decade there has been a tendency towards case-based learning as an instructional approach for students to learn medical problem-solving [13], [14]. To foster the development of expertise early in medical careers learning from authentic patient cases has been stipulated [15]. The key to successful learning of medical students seems to lie in the consequent process character of the cases [16]. Despite this empirical basis it remains hard to assess the verification if, when and how to foster medical students’ problem-solving skills. Even more, there is currently no established model in medical education to accurately describe the cognitive process of clinical problem solving. In order to educate with a resource-oriented instructional approach it is a prerequisite to first investigate the actual process of medical student's problem-solving.

When confronted with a problem, humans tend to take the same cognitive actions regardless of the content of the problem [17]. Cognitive actions could be defined as follows: the retrieval of the problem, the processing of the information, a formulation of the plan to tackle the task, carrying out the plan and an evaluation of the results. These cognitive actions have been thoroughly researched and are found in abundance known as action theoretic approaches in cognitive psychology [17], [18], mathematics [19], pedagogy [20], in medicine [21] and many other fields [22]. A medical problem-solving process including the underlying cognitive actions could be exemplified as follows: When a patient sees a doctor, the doctor recognizes or finds out about the symptoms of the patient (i.e. she complains about red urine), analyses these symptoms and generates differential diagnostic ideas (i.e. urinary tract infection). In order to get more information the physician asks further questions and performs further investigations (i.e. by examining the patient and carrying out a urine sample and a blood test). When presenting the patient to another physician, the doctor would summarize what he or she has learned so far from an inner representation of the patient (i.e. 57 year old female patient, hematuria since three days, no signs of an infection). This inner representation includes positive and negative findings and might as well contain differential diagnostic ideas (i.e. malignant tumour or glomerulonephritis). After an evaluation of the differential diagnoses, decisions about further steps would be reached and communicated to the patient. All models include the above mentioned cognitive actions with varying emphasis [17]. These cognitive actions serve as the foundation of the strategies of problem-solving within a field including medicine. A more adaptable and faster learning of clinical reasoning founding on the empirical verification of cognitive actions has been stipulated very recently [23], [24]. The model using typified objects (MOTmodel) comprehensively describes cognitive clinical reasoning process as suggested by experts. On the top-level of this hierarchically built model the experts agreed on the following processes: Identify early cues, determine the objectives of the encounter, categorize for the purpose of action, implement purposeful action and evaluate the results. All processes are interlinked and receive specific inputs and produce certain outputs thus representing the dynamic nature of the problem-solving process of experts. However, cognitive actions were not examined empirically among medical students. This is especially surprising as the development of medical students’ problem-solving skills could be fostered using knowledge about an optimum relation of cognitive actions. Furthermore, so far there is no evidence available that using certain cognitive action models predict successful case solutions.

The aim of this study was to empirically examine how medical students think clinically with the following objectives: (1) can the process of clinical problem-solving be described using the proposed cognitive actions; (2) can a specific pattern in case-based problem-solving be extracted using the relation of the proposed cognitive actions to each other; (3) is this pattern correlated with the diagnostic accuracy?

## Methods

### Operationalization of the Research Questions

The stated research questions were investigated in a laboratory setting with a controlled set of clinical content. A think-aloud method was used to be able to identify patterns and certain subcomponents of thinking. Paper-based cases with basic patient information and further on test-results were given to the subjects.

### Participants

Twenty-three medical students in their 4^th^ or 5^th^ year (female = 11) of two medical faculties volunteered (M = 23.9 years; range 20–34) to take part in the study. These years of the medical curriculum were chosen because the participants should have enough prior knowledge to solve clinical problems, but should not have experienced their final 6^th^ clinical year of full time electives to focus on the the problem-solving of the student. Furthermore these participants had finished their internal medicine curriculum. Written informed consent was obtained from all participants. This study was approved by the Ethical Committee of the Medical Faculty of LMU Munich. Participants received a small monetary compensation for their expenses.

### Operationalization of the Model

It has been criticized that action theoretic models might be useful for instructional purposes, but are not suitable to describe the real-life problem-solving processes [22]. To conduct empirical research, an analysis model was needed to concretize the task, most likely applicable to medical students and detailed enough not to miss fundamental cognitive actions. After a thorough literature review and comprehensive expert discussions the empirically tested model from Schoenfeld [25] was chosen as a starting point as it represents the widely used action theoretic models, with the following cognitive actions: *read*, *analyse*, *explore*, *plan*, *implement* and *verify*. Schoenfeld’s model was especially formulated for simple problem-solving dealing with a single problem, but not for complex problems [25]. Problems can be considered as complex where diverse and volatile goals have to be considered [17]. Medical problem-solving is complex problem-solving [17]. Thus, more cognitive actions needed to be defined to gain a comprehensive view. Therefore, the original Schoenfeld model was modified in the following way. The doctor needs an *inner representation* to cope with the complexity of the problems, the development of which is another cognitive action within the analysis model. With this inner representation of the problems, the doctor *evaluates* the different actions taken and *integrates* the results to finally come to a solution. This decision for a working diagnosis or for the final solution is another cognitive action in the analysis model. The here presented “modified Schoenfeld model for complex problem-solving” (further referred to as “modified Schoenfeld model”) consists of eight selective cognitive actions, dealing with the problems given: *Denomination*, *Analysis*, *Exploration*, *Plan*, *Implementation*, *Evaluation*, *Representation*, *Integration* (see table 1). This “modified Schoenfeld model” was used for the case sessions of a pilot study. The detailed subactions and contents of each cognitive action were observed, summarized and defined using qualitative research methods (qualitative content analysis, inductive category development, open coding process [26]). After several test codings, a fixed coding scheme was defined and applied to the whole sample of cases.

**Table 1:** Illustration and operationalized definition of the “modified Schoenfeld model for complex problem solving”.

Cognitive Action	Operationalized definition
Denomination	Retrieve information; read
Analysis	Analyse information; generate differential diagnostic ideas
Exploration	Associate, compare, vaguely propose strategies how to understand the problem
Plan	Generate plans, weigh up these plans against each other, decide on a plan
Implementation	State and justify one definite plan; request certain additional information and/or examinations
Evaluation	Verify or dismiss hypotheses with regard to new information or examination results; evaluative thinking
Representation	Inner representation of the case; statement of the situation as far as it is summarized in the mind of the student
Integration	Decision for one working diagnosis, differential diagnoses and/or therapy

### Course of the Study

**Figure 1:**
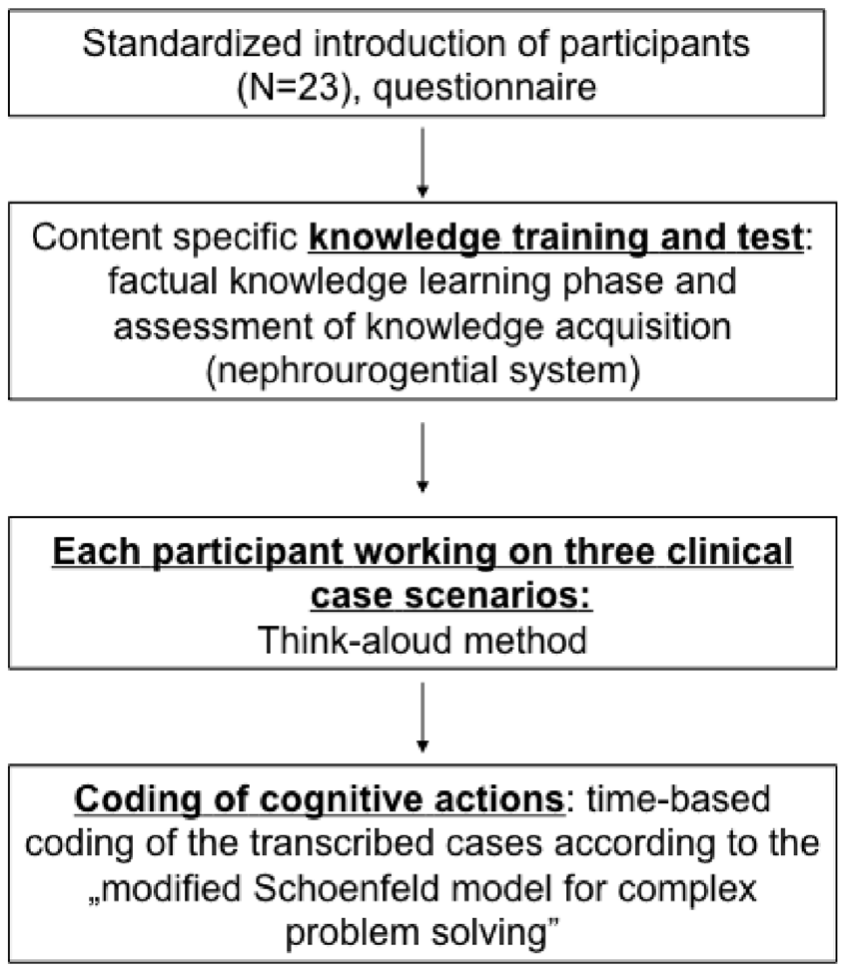
Overview on the course of the study.

Figure 1 shows that the study consisted of a controlled knowledge training, a subsequent knowledge test, and the paper-based clinical case-scenarios. Participants solved three cases in clinical nephrology with the think-aloud-method after three hours practising a standardized learning unit in the field of clinical nephrology. Recordings were transcribed and coded according to the “modified Schoenfeld model”. Codings were analysed for accuracy of the diagnosis. Learner characteristics were obtained by questionnaires.

#### Pre-study questionnaire

All participants filled out a questionnaire containing items about their socio-demographic data, gender and age as possible confounders. The reliability of this multiple-choice exam is very high (Cronbachs α = .957) [27]. The performance of participants in this exam was used as an indicator for general prior knowledge in medicine. The results of the questionnaire and all other obtained data were anonymized.

#### Knowledge training and test

Although all participants were in the advanced part of medical school and had all passed the internal medicine curriculum a pre-learning phase was established. The pre-learning phase involved an extensive 3-hour computer-based tutorial on clinical nephrology to account for content specificity [4]. This was to help ensure that all students were able to show their problem-solving strategy and ability because they had the knowledge needed for application of strategies. Upon completion, the students' retention of content specific medical knowledge was tested [13], [16], [28].

#### Clinical case scenarios

The three paper-based case scenarios with diagnoses within the field of clinical nephrology were real cases of the department of internal medicine adapted from experts with anonymized real supplemental material (i.e. lab values). After the transformation into paper-based scenarios, authenticity was additionally ensured through review by two content experts and one didactic expert. All cases were structured the same way, containing two or three pages describing the patient´s complaints and medical history. The results of the physical examination, blood tests, urine sample, ECG and ultrasound scan were each described on separate pages. The first case described a patient with hematuria due to glomerulonephritis. The second case concerned a patient with both the symptoms of acute renal failure as well as depression. The third case was on a patient with hypertensive crisis due to renal arterial stenosis. Students were not allowed to use secondary aids such as books or computers.

In a short practice exercise participants were instructed on the think-aloud method [29]. The students' task was to work on each case to show their problem-solving abilities with no other instructions being given than “please work on this case”. They were not explicitly asked to state a diagnosis. Only one single student and the test instructor were present in the room during the case elaboration. The test instructor sat behind the participant to avoid any diversion of thought [29]. The only interaction between the participant and instructor was when the instructor provided the next page of a case. Every case was interrupted after ten minutes, independent of whether the case was solved or not. While participants were working on the cases using the think-aloud method, they were audiorecorded.

### Data Analysis

All audio recordings (total time of 13∶05 hours) were transcribed and coded using the model described above. For technical reasons, three tapes were not completely evaluable and 66 of 69 cases were analyzed. The standard qualitative content analysis by Mayring [26] was used as method to assess, code and analyse the process of thought, as it also yields very detailed quantitative data in consecutive analysis. It uses models with several categories for the coding of a text. In this study, the cognitive actions were used as categories. A section of text matching a particular cognitive action was determined as an episode. One text section could be coded as more than one episode, when different cognitive actions took place at the same time. Subsequently, the codings were marked as time-sections in the transcription software “f4” (f4 2011, Dr. T. Dresing, http://www.audiotranskription.de) and then exported to Microsoft Excel 2010 (Microsoft, 2010). For further analysis the statistical environment “R” was used (http://www.r-project.org/). A predefined alpha level set at p<0.05 was used for all tests of significance. Graphical illustrations were processed as the percentage of time spent on one action relative to the overall time. Although the cognitive actions of the model were described qualitatively, this was the basis for a quantitative analysis and graphical illustration of the results.

As quantitative dependent variables the frequencies of cognitive actions were analysed, as well as the length of the episodes.

The accuracy of diagnosis was established in a binary form (correct or not correct) as a dependent variable. Chi-squared tests were used to verify the relationship of dependent variables to all dichotomous participant variables, while Pearson correlation was used for all continuous dependent variables to correlate them to previously obtained participant data. Chi-squared tests were processed in SPSS 20.0 with a predefined alpha level set at p<0.05.

One investigator (R. E.) coded all transcripts. A second rater coded more than 10% of the transcripts. Based on the coded time, the interrater coefficient analysed with Cohens kappa was κ = .935. Based on the coded text, the interrater coefficient was κ = .884.

## Results

The “modified Schoenfeld model for complex problem-solving” in medicine enables us to describe the cognitive actions of medical students. The times-on-task participants spent overall on each of the eight cognitive actions are shown in table 1. Most time was spent on the cognitive actions *Denomination* and *Analysis.* The frequencies of the episodes overall showed a similar distribution with minor distinctions. Action *Denomination* and *Analysis* have mainly long episodes (M_Denomination_ = 45sec ±1.74, M_Analysis_ = 51sec ±2.34). Action *Implementation* often consists of short episodes (M_Implementation_ = 19.10sec ±1.11), so the percentage in terms of frequencies is higher than the percentage in terms of session-time (as illustrated in table 2).

**Table 2:** Time-on-task: Distribution of the cognitive actions (all cases, n = 66).

Cognitive action	Denomi-nation	Analysis	Exploration	Plan	Implemen-tation	Evalu-ation	Represen- tation	Integra-tion	All cases
**time (relative %)**	360.5 min (36.76%)	281.5 min (28.71%)	40.4 min (4.12%)	53.8 min (5.49%)	75.4 min 7.69%)	85.5 min (8.76%)	45.5 min (4.65%)	37.3 min (3.81%)	**980.4 (100%)**
**Frequency of episodes (relative %)**	474 (32.09%)	330 (22.34%)	62 (4.2%)	77 (5.21%)	237 (16.04%)	159 (10.76%)	79 (5.35%)	59 (3.99%)	**1.477 (100%)**

**Figure 2:**
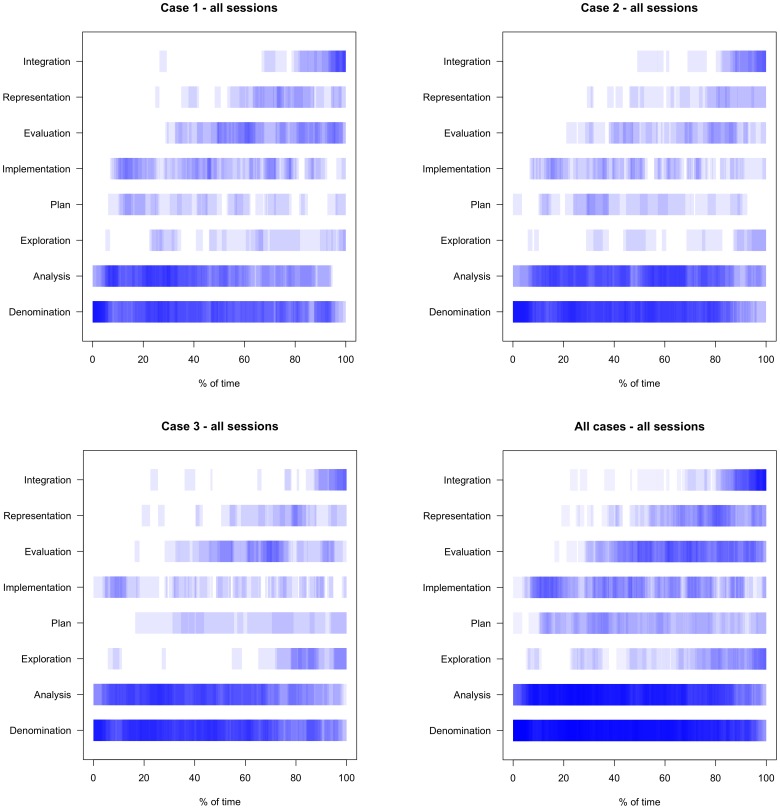
Time-line graphs of all participants of each clinical case, and time-line graph of all clinical cases (Figure 2 a to d from upper left to lower right corner). It shows the distribution of cognitive actions over time. The darker the blue is presented, the more case elaborations are containing this action at this part of the process.

Figure 2 shows how the cognitive actions were distributed over time. All elaborations are presented separately for each of the three cases (Fig. 2a–c) and aggregated for all three cases (Fig. 2d). The case elaborations of all participants were mapped onto each other. As the figure shows, *Denomination* and *Analysis* were spread over the entire case elaboration, equally *Plan* and *Implementation*. The cognitive actions *Evaluation*, *Representation* and *Integration* were not present at the beginning and emerged during the case elaboration in this order. This pattern evolved for each of the three cases in a similar way (compare Fig. 2a–c).

### Elucidation of a “*Complete Model* Pattern”

**Figure 3:**
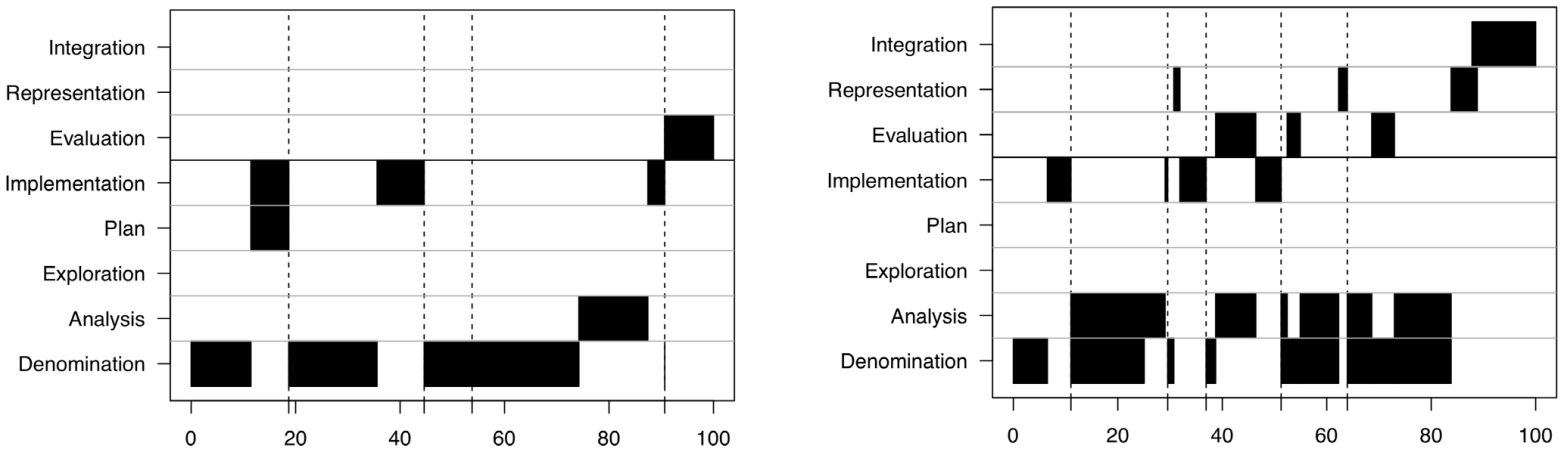
Time-line graphs of case elaborations with incomplete (3a) and *complete model* (3b). When the case elaboration included also the higher loops of the actions Evaluation, Representation and Integration these case elaborations were labelled a “complete model”. If the actions Evaluation, Representation and Integration were in another order or only single actions were coded, the case elaboration was labelled “incomplete”.

In most individual case elaborations, two or three cognitive actions took place at the same time. Mostly this was *Analysing* or *Evaluating* while *Denominating* (44% of coded categories). To identify patterns in the case elaborations, the time-line graphs of the single cases were analysed. The analysis revealed a typical reproduced sequence how the participants traversed through the cognitive actions: they mostly started with *Denomination*, progressed through *Analysis* (or sometimes *Exploration*) to *Implementation* (or more rarely *Plan*). The obtained new information, due to the requests of the cognitive action *Implementation,* are then read and *denominated*, and another loop starts from the beginning of this sequence again. We keyed this sequence, which was found in every case elaboration, a “lower loop” (M_loop_ = 3.18 loops/case ±1.46). The most widely used sequence of cognitive actions in the lower loops was *Denomination*, *Analysis*, *Implementation*, *Denomination* (116 of 210 loops; 55%). The actions *Evaluation*, *Representation* and *Integration* did also show a typical sequence in more than half of the case elaborations (37/66; 56%). This sequence was called “higher loop”. The sequence began with Evaluation and optionally *Representation*, followed or closed by *Integration*. As only explicitly stated representations were coded, *Representation* was considered to be optional. When the case elaboration included both, the lower loops as well as higher loops of the actions *Evaluation, Representation* and *Integration* these case elaborations were labelled a “*complete model*” (37/66; 56%). If the actions *Evaluation*, *Representation* and *Integration* were in another order or only single actions were coded, the case elaboration was labelled “incomplete” (29/66; 44%). The *complete model* was equally distributed over the three given paper-based cases, with a lower frequency in the third case (first case: 14/23; 61%, second case: 13/22; 59%, third case: 10/21; 48%). Figure 3 shows representative case examples each for a *complete* and an *incomplete model*.

### The Complete Model Pattern is Significantly Correlated with the Correct Diagnostic Case Solution

Neither socio-demographic data of the participants (age, year of studies), nor prior knowledge (grades of PME as general prior knowledge, assessment of the learning phase in the field of clinical nephrology) were related to the completion of the model, analysed with Pearson correlation. As well, the dichotomous variables of sex and practical experience were not related to the completion of the model, analysed with Chi square test. Previous knowledge is not correlated with the *complete model* or for the correct solution in this setting with this level of knowledge in clinical nephrology.

The correct solution was obtained in 27 of all cases (27/66; 41%), the incorrect solution or no solution in the majority of the case elaborations (39/66; 59%), respectively. Out of the 37 cases with the *complete model*, the correct solution was reached in 24 cases (24/37; 64%). In contrast, out of the 29 cases with the *incomplete model*, the correct solution was reached in 3 cases only (3/29; 10%) (see table 3). The *complete model* was a strongly correlated with the correct solution. (Chi-squared test, p<.0001; phi coefficient [mean square contingency coefficient] φ = 0.55).

**Table 3:** Frequencies of the incorrect and correct solution relative to the completion of the model.

	Incorrect solution	Correct solution	
Incomplete model	26/29; 90%	3/29; 10%	**29 cases; 44%**
Complete model	13/37; 35%	24/37; 64%	**37 cases; 56%**
	**39/66; 59%**	**27/66; 41%**	

## Discussion

The aim of the study was to empirically verify the process of complex problem-solving among medical students. The first objective was to determine whether the process of problem-solving can be described using the cognitive actions in the proposed “modified Schoenfeld model”. The results indicate that it is possible to describe the process of problem-solving using this model. More specifically, it was found that all medical students used the following cognitive actions: *Denomination*, *Analysis*, and *Implementation*. When dealing with the cases, the medical student participants spent 73% of the session time with these relatively basic cognitive actions. Further, the results yield that the students spend less time on the actions *Exploration* and *Plan*. Furthermore, the cognitive actions of *Evaluation*, *Representation* and *Integration* were found only in a subset of the students. On average, students spent only 17% of the total session time on these higher cognitive actions.

The second objective of this study was to assess whether certain patterns can be extracted in the distribution of the actions over the duration of the case sessions. In our analysis, certain repeating patterns were found. Among all students the pattern of *Denomination* to *Analysis* and to *Implementation* could be found and was called a lower loop. This finding is consistent with the loops in the problem-solving process of medical doctors as described by Barrows and Tamblyn [21]. The higher cognitive actions (higher loops) could be coded in 56% of all cases. Solving a case with both, the lower loops and the higher loops was defined as the *complete model pattern*. The overall process of the case elaboration revealed a dynamic and complex sequence of actions with various lengths and often rapid switching between the different actions. The non-sequential workflow observed in the case elaboration in this study can be assumed to be necessary to cope with the complexity of the problems (as described in action theoretic approaches [17]).

The third objective was to reveal whether the identified pattern is associated with the solution of the case. The *complete model pattern* was significantly correlated with a higher frequency of the correct solution (φ = 0.55). It appeared that the higher cognitive actions *Evaluation*, *Representation* and *Integration* were crucial for successful problem-solving. A reason for this finding might be that these cognitive actions exceed the other five cognitive actions with regard to their cognitive complexity needed to execute these actions as they require the ability for abstract thinking. For problem-solving of complex medical cases by medical students the quality of process was strongly associated with the quality of product in our study (cf. van Gog [16]). Furthermore, this finding can be explained through the attributes of complex problem-solving [17]. Here, working on a case does not happen in a sequential order but rather in a dynamic and complex process where transitions from one action to another back and forth are necessary due to multiple problems and aims which change over time. Therefore, the ability to build an inner representation from the case information and its evaluation enabled the students to reach the correct solution. Surprisingly, the extent of general prior medical knowledge (PME) was neither related to the complete model pattern nor to the correct solution of the case. Therefore, this result suggests that the completion of the model is independent from the person. The question remains whether the higher cognitive actions are a predictor for diagnostic accuracy or rather a prerequisite. Furthermore, the fulfilment of the model could not simply be attributed to students with higher grades. According to content specificity, knowledge in a certain field is a prerequisite for the strategies applied. Although content specificity was controlled through the learning phase, the subjects did not consistently use or not use the *complete model* nor did the grades of the assessment after the learning phase relate to the use of the complete model. This result indicates that the cognitive actions described could be indeed fundamental abstractions, that they are not completely based on content specificity. Further research should clarify the counterintuitive finding regarding general prior knowledge (as tested with the PME). For example, the relation of knowledge types (factual knowledge, conceptual knowledge and procedural knowledge [30], [16]) and meta cognitive knowledge and regulation [31], [32] to the cognitive actions, the completion of the model and the solution of the case should be investigated.

The implementation of the model into a cognitive architecture (i.e. ACT-R; adaptive control of thought–rational) would be interesting. Cognitive architectures have also been used to model the problem-solving processes of mathematicians and then implemented to foster the mathematical problem-solving of high-school students [33]. Although medical problem-solving is different from mathematical problem-solving a transfer of this application seems highly desirable. Additionally the model could be used as a tool for expertise research in medical problem solving and for research on specific biases of decision making of physicians [34].

### Potential Applications for Medical Education

There is an abundance of educational models using sequential steps [17], [35]. For clinical reasoning, the most common models are problem-based learning [21], [36], [37] or worked examples [13], [16], [38]. These models were designed for instructional purposes of core curriculum knowledge but have been criticized to be unsuitable for a description of realistic free individual medical problem-solving as happens in daily clinical work [22]. The findings in this study demonstrate that the proposed model is well-suited to describe realistic free individual medical problem-solving of medical students. The value of the model consists in its capacity to enable one to trace back the cognitive steps students take during the medical problem-solving process, independent of the correct solution. This is different from current educational strategies where the focus lies on the correct solution rather than the process towards the correct solution (cf. van Gog [16]). One educational application which can be drawn from this study is the necessity to foster higher level reasoning (evaluation, representation and integration) during case elaboration. This could for example be applied by supporting students to express a verbal representation during their individual problem-solving process. Furthermore, training students to present their patients also may foster higher level thinking; research is needed to verify how this might work. This study showed that the majority of the students were already able to think on the higher-level. Therefore, instruction and encouragement alone could be a resource-oriented approach [39]. In case-based learning, worked examples could advance students’ learning to higher-level thinking as especially *Integration* could be fostered. With the model it is now possible to evaluate instructional strategies regarding their underlying cognitive actions. However, before the model should be used in this way it is important to understand why the students chose certain cognitive actions and did not choose others. Future studies on this subject could be stipulated by selection strategy research (i.e. [40]).

### Limitations of the Study

The qualitative design, the data preparation, as well as the analysis made it necessary to include a limited number of participants and a limited number of cases and domains per participant, respectively. On the other hand, qualitative research chooses to rather focus on carefully constructed valid measures (over thirteen hours of transcribed, coded and analysed material) than on less meaningful yet reliable measures, and for a qualitative study, the sample is relatively large. The composition of participants in the study was selected by stratification in groups regarding to their years of study, age and sex. However, the findings support that the completion of the model and solution of the cases were not linked to the participants at all. A natural limitation created by the think-aloud method is that only what is expressed verbally can be analysed, coded and interpreted. Furthermore, the model is rather complex and not easy to code. The eight cognitive actions were chosen in order not to miss a cognitive action. For further investigations, it could be useful to work with a simplified model by fusing both the cognitive action of *Analysis* and *Exploration* as well as *Plan* and *Implementation*.

Our model represents one way of approaching the cognitive processes behind clinical reasoning. Our model was drawn inductively from various models and pilot study data. Certainly other existing models have been proposed that could also fit. Recently elaborated and extensive modelling did find steps similar to our proposed model [23]. Nonetheless, to our knowledge our study represents the first empirical verification of a model to describe the process of individual medical problem-solving among medical students and it strongly suggests a link between higher cognitive actions and successful case solutions.

### Conclusions

The model used in this study investigates the complex and dynamic nature of the medical problem-solving process. We have investigated and validated a first model to describe the cognitive actions during problem-solving of clinical medical students. This provides the platform for further research especially for the evaluation of novel instructional methods that intend to foster clinical reasoning.
